# Cognitive behavioral interventions for depression and anxiety in adults with neurological disorders: a systematic review and meta-analysis: reply to Zhou et al

**DOI:** 10.1017/S0033291725000613

**Published:** 2025-03-24

**Authors:** Milena Gandy, Thomas Woldhuis, Wendy Wu, Marette Youssef, Madelyne A. Bisby, Blake F. Dear, Andreea I. Heriseanu, Amelia J. Scott

**Affiliations:** School of Psychological Sciences, Macquarie University, Sydney, Australia

We thank Zhou and colleagues for their encouraging comments and review of our recent meta-analyses. We would like to share some general comments and thoughts in response.

One key point raised by Zhou, Zheng, and Tian (2025) was the potential for different effects if we had used fixed-effect models in our main analysis. First, we provided a clear rationale for our decision to use random-effects models (Gandy et al., 2024), which are the most commonly employed in meta-analyses of psychological interventions (Cuijpers, 2016). This decision was based on both statistical and methodological heterogeneity across studies, including differences in populations, interventions, settings, and other features (Dettori, Norvell, & Chapman, 2022; Serghiou & Goodman, 2019). Notably, Zhou et al. themselves pointed out this heterogeneity.

In this context, the rationale for using fixed-effects models remains unclear, and we would be interested in Zhou et al.’s reasoning as it was not provided in their commentary.

Importantly, we note that the estimate from Zhou et al.’s fixed-effect model produced a nearly identical result, particularly when considering the confidence intervals around the point estimates. Table 1 further illustrates this by showing that when using all available study arms, the differences between fixed-effects and random-effects models were negligible. Therefore, even if we had used fixed-effects models, our core conclusions would remain unchanged.

**Table 1.** Comparison between random and fixed-effects models

	Random effects	Fixed effects
Anxiety (k = 29)	*g* = 0.38, 95% CI: 0.29–0.48	*g* = 0.37, 95% CI: 0.29–0.45
Depression (k = 57)	*g* = 0.45, 95% CI: 0.35–0.54	*g* = 0.41 95% CI: 0.35–0.46

CI = Confidence Interval

Another key point Zhou et al. made was about the variability of outcome measures, which needed to be included in the analysis. We agree with this point, which is a challenge common to nearly all meta-analyses of psychotherapy. In fact, this was a key reason we conducted moderator analyses based on outcome measure selection, providing effect size estimates for each measure type. We do not see a feasible alternative approach to addressing this issue, however, we would appreciate any recommendations that Zhou et al. might have.

Similarly, as Zhou et al. note, differences between active and inactive control groups can influence meta-analysis findings. This is precisely why we conducted a moderation analysis based on the control group type. Interestingly, our results indicate that the control group type moderated effects for depression but not anxiety. Importantly, we discuss effect size estimates based on control group selection within our Discussion section. Again, we do not see a feasible alternative approach to addressing this common issue. We agree that future network meta-analyses would provide valuable insights for the field, particularly as more trials are published.

The authors propose using the Grade of Recommendations Assessment, Development, and Evaluation (GRADE) system, which is used to assess the quality of evidence and strength of recommendations in healthcare and clinical practice guidelines. However, GRADE is not typically applied to meta-analyses focused on the estimation of treatment effects (Cuijpers et al., 2023; Hedman-Lagerlöf et al., 2023). In line with standard practice for meta-analyses of randomized controlled trials, we assessed the quality of individual studies and conducted sensitivity analyses to determine whether study quality influenced outcomes. Our aim was not to develop clinical guidelines but to evaluate the state of the evidence and quality of available trials, which we assessed using the Cochrane Risk of Bias tool, a standard approach in psychotherapy meta-analyses. It is difficult to comment on the authors’ overall GRADE assessment, as they do not provide data on their evaluation of individual studies.

Finally, we thank the authors for their kind appreciation of our work. We share their view that the ultimate goal of research in this area is to guide the optimization of interventions and improvement of outcomes for individuals with neurological disorders and mental health challenges, in alignment with their conclusions.
